# The protective effect of kaempferol on high glucose-stimulated renal tubular epithelial cells

**DOI:** 10.1186/s12882-025-04404-9

**Published:** 2025-08-20

**Authors:** Xiao-Cui Jiao, Ying Li, Di Wu, Xue-Guang Zhang, Fei Hou

**Affiliations:** 1 Department of Nephrology, Capital Medical University Electric Teaching Hospital, Beijing, 100073 China; 2 Department of Nephrology, Hebei Medical University Third Hospital, Shijiazhuang, 050051 China

**Keywords:** Kaempferol, High glucose, Renal tubular epithelial cell, Oxidative stress, Apoptosis, Sirtuin3

## Abstract

**Background:**

The oxidative stress and apoptosis of renal tubular epithelial play an important role in the progression of diabetic nephropathy. Blocking oxidative stress and apoptosis of renal tubular epithelial could be a novel therapeutic target for diabetic nephropathy. Kaempferol (KMP), a natural phytoestrogen and common dietary flavonoid, has various biological effects including anti-oxidation, anti-apoptosis and anti-inflammation. KMP has protective effect against oxidative stress-related diseases, such as ischemia-reperfusion induced myocardium injuries, osteoporosis, obesity and so on. In our research, we observed the influence of KMP on high glucose (HG) cultured HK-2 cells and explored its mechanisms from the aspect of oxidative stress and apoptosis.

**Methods:**

To find out the safety and effective concentration of KMP in our experiment, cell viability under different concentrations was detected using the MTS method. The protein and mRNA expression of SOD2 and catalase were detected by Western blot and Real-time PCR; the protein expression of Sirt3, Bax, Bcl-2, cleaved-caspase3, Akt, p-Akt, FoxO3a, p- FoxO3a were detected by Western blot; the ROS level in cellular was detected by cell flow cytometer.

**Results:**

We found that HK-2 cells stimulated with both 10µM KMP and HG exhibited higher viability compared to those stimulated by HG only. Incubation with KMP could reverse the undesirable effects of HG on SOD2, catalase, cleaved caspase-3, Bax/Bcl-2 ratio and the generation of ROS. Furthermore, Western blot and Real-time PCR results showed that the expression levels of Sirt3, p-Akt/Akt ratio and p-FoxO3a/FoxO3a ratio were markedly increased in the KMP plus HG group compared to the HG group. Furthermore, downregulating Sirt3 expression in HK2 cells impairs the protective effect of KMP, leading to a reduction in HK2 cell viability and an elevation in ROS levels.

**Conclusion:**

In summary, KMP could alleviate HG-induced oxidative stress and apoptosis, and its cytoprotection is associated with Sirt3 expression and the activation of ROS-sensitive Akt/FoxO3a signaling pathway.

**Supplementary Information:**

The online version contains supplementary material available at 10.1186/s12882-025-04404-9.

## Introduction

Diabetic nephropathy (DN) is the main cause of chronic kidney disease and end-stage renal disease in the United States [1]. Large numbers of studies have shown that hyperglycemia-induced oxidative stress play an important role in the progression of DN. Hyperglycemia not only increases mitochondrial reactive oxygen species (ROS) generation but also destroys anti-oxidative mechanisms through glycation of the scavenging enzymes [2]. Mitochondrial oxidative stress can cause tubular injury, including tubular apoptosis and tubular hypertrophy. Tubular injury is a major pathological change in DN and contributes to renal function deterioration [3]. ROS generated due to hyperglycemia also activates several apoptosis-related intracellular signaling pathways including PI3k/Akt/FoxO [4]. Therefore, inhibiting oxidative stress and apoptosis in tubular could be a novel therapeutic target for DN. Attractive therapeutic alternatives are emerging. Howerer, these agents do not block the progression of DN completely [5, 6]. Thus, it remains a critical issue to search for effective therapies to dampen the progression of DN.

In recent years, natural phytoestrogens (plant-derived polyphenolic non-steroidal compounds) have drawn considerable attention for its protective and therapeutic effects on cardiovascular diseases, osteoporosis, diabetes and obesity [7]. Kaempferol (3,4',5,7-tetrahydroxyflavone, KMP), a natural phytoestrogen and common dietary flavonoid, exists in many plants including grapefruit, tea, broccoli and so on [8]. KMP has various biological effects including anti-oxidation [9–11], anti-apoptosis [12–14] and anti-inflammation [15–17]. KMP has been demonstrated to alleviate insulin resistance via its anti-inflammation effect in type 2 diabetic rats [18]. KMP has also been reported to alleviate oxidative stress and apoptosis in ischemia-reperfusion injured myocardium [12]. Nevertheless, the protective effects of KMP against DN and the exact mechanisms have not been studied in depth.

Sirtuins, a conserved family of nicotine amide dinucleotide (NAD+)–dependent histone deacetylase, have been proven to be capable of extending life span [19]. Sirtuin3 (Sirt3), one of the seven mammalian homologs of sirtuins, is mainly located in mitochondrial inner membrane. Sirt3 has been reported to modulate mitochondrial function and thermogenesis by regulating the gene expressions and activities of enzymes related to many cellular processes such as mitochondrial respiratory chain, antioxidant defense, tricarboxylic acid cycle and apoptosis [20, 21]. Sirt3 regulates protein deacetylation, which is important for mitochondrial metabolism, cell survival and longevity. There has been an explosive growth of studies on the protective effects of Sirt3 in cardiovascular diseases, diabetes, kidney diseases, neurodegenerative disorders and so on [22]. H. Cimen et al. reported that the expression of Sirt3 in mitochondria could be increased by KMP treatment, thus decreasing acetylation of the SdhA subunit and increasing Complex II activity [23]. However, study about the relationship between KMP and Sirt3 is rare.

In this research, we observed the influence of KMP on high glucose (HG) cultured human kidney 2 (HK-2) cells and explored its mechanisms from the aspect of oxidative stress and apoptosis.

## Materials and methods

### Cell line and reagents

HK-2 cells were purchased from American Type Culture Collection (Manassas, VA, USA). D-glucose, mannitol, 2′,7′-dichlorodihydrofluorescein diacetate (DCHF-DA) and KMP were purchased from Sigma (St. Louis, MO, USA). Sirt3 and catalase antibody were purchased from Proteintech (Chicago, IL, USA). Antibody for superoxide dismutase 2 (SOD2) was purchased from Abcam (Cambridge, UK). Antibodies for Akt, p-Akt (Ser 473), Bax, Bcl-2, Forkhead box protein O3A (FoxO3a), p-FoxO3a and cleaved caspase-3 were purchased from Cell Signaling Technology (Beverly, MA, USA). FuGENE HD Transfection Reagent, reverse transcription system, qPCR Master Mix, MTS cell Proliferation and Cytotoxicity Assay Kit were purchased from Promega (Madison, WI, USA). TRIzol reagent, NE-PER nuclear and cytoplasmic extraction kit were purchased from Thermo Fisher (Carlsbad, CA, USA). Polyvinylidene difluoride (PVDF) membranes were purchased from Millipore (Billerica, MA, USA). Lipofectamin 3000 transfection reagent were collected from Thermo Fisher Scientific Inc. (USA). Sirt3 short interfering ribonucleic acid (siRNA) was designed and synthesized by Gemma company (Shanghai, China).

### Cell culture and treatment

The proximal tubular cell line, HK-2 cells, were cultured in DMEM supplemented with 10% fetal bovine serum, 100 U/ml penicillin and 100 µg/ml streptomycin in a 95% air plus 5% CO_2_ atmosphere at 37℃. When the cells growed 60%-80% confluence, they were stimulated with either normal glucose medium (NG, 5.6mM D-glucose), high glucose medium (HG, 30 mM D-glucose), osmotic control medium (M, 5.6 mM D-glucose plus 24.4 mM D-mannitol), KMP-contained medium at different concentrations (0.01 µM, 0.1 µM, 1 µM, 5 µM, 10 µM, 15 µM, 20 µM, 50 µM), KMP plus HG medium (HG + KMP) or KMP plus HG medium plus Sirt3 siRNA at indicated time points.

### MTS method

The HK-2 cells at logarithmic growth phase were digested by trypsin, adjusted to the concentration of 2 × 10^4^ cells/mL, inoculated into 96-well cell culture plates (100 µL per well) and then cultured at 37 °C for 24 h. When the cells growed 60–70% confluence, they were treated with various concentrations of KMP (0.01 µM, 0.1 µM, 1 µM, 5 µM, 10 µM, 15 µM, 20 µM, 50 µM), high glucose and Sirt3 siRNA for 48 h. Cell proliferation was measured by MTS assay according to the manufacturer’s instructions. Simply, the supernatant of each well was removed, then 20 µL MTS reagent (5 mg/mL) was added to each well. After incubation for 4 hours, cell viability was analyzed and recorded at 490 nm using a microplate reader (GE, USA).

### Quantitative real-time PCR

Total RNA of HK-2 cells was extracted using TRIzol reagent according to the manufacturer’s instructions. The cDNA was synthesized using a cDNA synthesis kit. Primer sequences for all the genes were designed using Primer5.0 for real-time PCR. Primer sequences were as follows:

β-actin, forward 5′-ACACGGACAGGATTGACAGA-3′ and reverse 5′-GGACATCTAAGGGCATCACAG-3′; 

Sirt3, forward 5′-TGCCAGCTTGTCTGAAGCA-3′ and reverse 5′-GTCCACCAGCCTTTCCACA-3′;

SOD2, forward 5′-GCGGCCTACGTGAACAACCTGAAC-3′ and reverse 5′-CCGTTAGGGCTGAGGTTTGTCCA-3′;

catalase, forward 5′-GCTGGTTAATGCAAATGGGGAGGC-3′ and reverse 5′-CCTGGGAAAGTCTCGCCGCATCT-3′.

The qPCR Master Mix was used for real-time PCR to quantify the expression of cDNA. PCR was performed on the Viia7 Real-Time PCR System (Applied Biosystems). Data were analyzed using the 2^–ΔΔCT^ method and presented as fold changes relative to mRNA expression levels of β-actin as an internal control.

### Protein extraction and western blot

Whole cell protein was extracted using RIPA lysis buffer containing protease and phosphatase inhibitor. Thirty micrograms of protein was separated by 10%-12% SDS-PAGE gel and then transferred to PVDF membranes by electrophoresis. The membranes were incubated overnight with the indicated primary antibodies. The protein bands were visualized with corresponding horseradish peroxidase(HRP)-conjugated secondary antibodies and ECL detection reagent by the Odyssey Fc System (LI-COR, USA). The densitometry of the protein bands was measured using ImageJ software.

### Intracellular ROS detection

The HK-2 cells were cultured in 6-well plates and treated as previously described. Then they were washed three times with PBS. Next, they were incubated with 10µM fluorescence probe DCHF-DA in PBS at 37 °C for 30 min, washed in order to remove the residual probes, trypsinized, and suspended in PBS. The intracellular ROS were measured with a flow cytometer (BD Immunocytometry Systems, Franklin Lakes, NJ, USA). The results were analyzed by FlowJo software.

### siRNA plasmid transfection

The HK-2 cells were inoculated into a 6-well plate with a density of 1*10^5^ cells/well. After adherence, Sirt3 siRNA plasmid was transfected with Lipofectamine 3000, according to the instruction. After the transfection with siRNA plasmid for 24 h, the levels of Sirt3 gene and protein were detected.

### Statistical analysis

The data were analyzed using the SPSS software package (IBM SPSS Statistics, Chicago, IL, USA). The experimental results are expressed as the mean ± standard deviation (SD) from three independent experiments. Comparisons between groups were performed with one-way ANOVA (three or more groups). The biological interaction between HG and KMP or between KMP and Sirt3 knock-down were tested by two-way ANOVA. Differences were considered statistically significant if *P* < 0.05.

## Results

### Effects of KMP on HK-2 cell viability in the absence or presence of HG

**Fig. 1:**
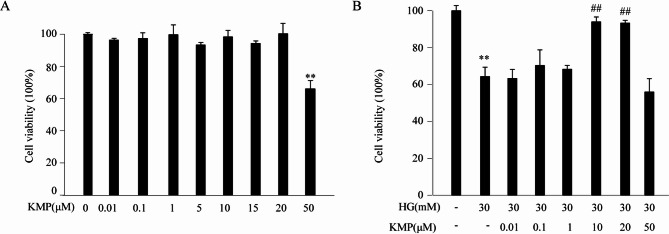
Effects of KMP on the viability of HK-2 cells. **A**: Cytotoxic effect of KMP on HK-2 cells was assessed with MTS assay(*n* = 3). **B**: Effect of KMP on HG-induced repression of cell viability was analyzed with MTS assay(*n* = 3). Cells were cultured with different concentrations of KMP in the absence or presence of 30 mM HG for 24 h. The values presented are the means ± SD of three independent experiments. HG: 30 mM D-glucose; KMP: 10 µM kaempferol ^**^P < 0.01 versus control group. ^##^*P* < 0.01 versus HG group

To test the cytotoxicity of KMP on HK-2 cells, cell viability was assessed through MTS method. As shown in Fig. 1A, KMP had no significant effect on cell viability at the concentrations of 0.01 µM, 0.1 µM, 1 µM, 5 µM, 10 µM, 15 µM and 20 µM. However, compared to the control group, cell viability decreased to 66% when the concentration of KMP reached 50 µM. This indicated that 50 µM KMP significantly inhibited the proliferation of HK-2 cells (Fig. 1A). In addition, we detected the effect of KMP on HG-induced inhibition of cell viability. In Fig. 1B, compared with the control group, cell viability decreased significantly following stimulation with high concentration D-glucose for 48 h. Co-stimulation of 10 µM KMP with HG increased cell viability compared to HG only (94 ± 2.6 vs. 64.3 ± 5.1) (Fig. 1B). These results suggested that KMP could reverse HG-induced cytotoxicity. Taken together, the concentration of 10 µM was selected for KMP in subsequent experiments.

### KMP protects against HG-induced oxidative stress

**Fig. 2:**
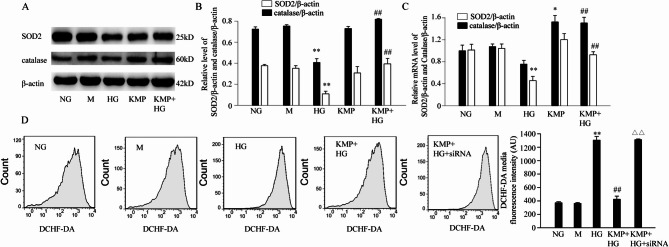
KMP reduces HG-induced oxidative stress. **A** and **B**: The protein expression levels of SOD2 and catalase were detected by Western blot (*n* = 3). **C**: The mRNA expression levels of SOD2 and catalase were detected by RT-qPCR (*n* = 3). **D**: Intracellular ROS levels in different groups were detected by flow cytometry (*n* = 3). The values presented are the means ± SD of three independent experiments. NG: 5.6 mM D-glucose; M: 5.6 mM D-glucose plus 24.4 mM D-mannitol; HG: 30 mM D-glucose; KMP: 10 µM kaempferol; KMP + HG: 10 µM kaempferol + 30 mM D-glucose. KMP + HG + siRNA: 10 µM kaempferol + 30 mM D-glucose + Sirt3 siRNA. ^*^P < 0.05 versus NG group. ^**^P < 0.01 versus NG group. ^##^*P* < 0.01 versus HG group. ^△△^*P* < 0.01 versus HG + KMP group

To assess the anti-oxidative stress effect of KMP under HG environment, the protein and mRNA levels of SOD2 and catalase were detected by Western blotting and quantitative real-time PCR respectively. The results revealed that 10 µM KMP plus HG increased the protein and mRNA levels of SOD2 and catalase compared with HG only (SOD2 protein 0.39 ± 0.05 vs. 0.11 ± 0.03, SOD2 mRNA 0.93 ± 0.05 vs. 0.46 ± 0.08, catalase protein 0.82 ± 0.007 vs. 0.41 ± 0.04, catalase mRNA 1.5 ± 0.1 vs. 0.76 ± 0.07) (Fig. 2A, B and C). In addition, we studied the effect of KMP on radical scavenging by quantitative evaluation of the changes of hydroxyl radicals through flow cytometry. As shown in Fig. 2D, intracellular ROS levels were markedly increased in HG group (1304.67 ± 55.53) compared with NG group (372.67 ± 14.57). The treatment of 10 µM KMP could significantly reverse HG-induced ROS accumulation (424.67 ± 47.06). As an osmotic control, mannitol did not affect intracellular ROS production.

### The effect of Sirt3 on the protective role of KMP against HK2 cells under high-glucose conditions

Next, we explored whether Sirt3 is involved in the protective effect of KMP on HK2 cells under high-glucose conditions. The HK-2 cells were divided into NG group, M group (mannitol group), HG group(high glucose group), HG plus KMP group(high glucose plus 10 µM KMP), HG plus KMP plus siRNA group(Sirt3 siRNA group). Co-stimulation of 10 µM KMP with HG increased cell viability compared to HG only (94 ± 2.6 vs. 64.3 ± 5.1). However, Sirt3 siRNA treatment significantly decreased cell viability (*P* < 0.05) in the HG plus KMP group (Table 1), suggesting that the cell viability protective effect of KMP was dependent on Sirt3 in HK2 cells. Additionally, we also observed the effect of silencing Sirt3 expression on intracellular ROS production. As shown in Fig. 2D, the treatment of 10 µM KMP could significantly reverse HG-induced ROS accumulation (424.67 ± 47.06). Compared with HG plus KMP group, intracellular ROS levels were markedly increased in Sirt3 siRNA group (1311 ± 10.82).

**Table 1:** Co-treatment of Sirt3 siRNA and KMP on HK-2 cell viability with high glucose

Groups	Cell viability(%)
NG	99 ± 1
M	98.8 ± 1
HG	65 ± 5**
KMP + HG	94.7 ± 2.08^##^
KMP + HG + siRNA	66.3 ± 2.08^∆∆^

Cells viability were analysed in different groups with MTS assay. The values presented are the means ± SD of three independent experiments. NG: 5.6 mM D-glucose; M: 5.6 mM D-glucose+ mannitol (24.4 mM); HG: 30 mM D-glucose; KMP: 10 μM kaempferol; KMP + HG: 10 μM kaempferol + 30 mM D-glucose. KMP+HG+siRNA: 10 μM kaempferol + 30 mM D-glucose + Sirt3 siRNA. Values are expressed as means ± SD. ^*^ P<0.05 vs. NG group. ^**^ P < 0.01 vs. NG group. ^##^ P < 0.01 vs. HG group. ^∆∆^P<0.01 versus HG + KMP group

### KMP attenuates HG-induced apoptosis in HK-2 cells

**Fig. 3:**
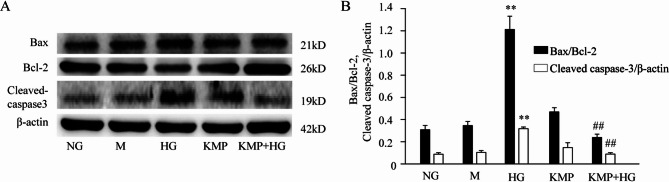
Effect of KMP on HG-induced apoptosis in HK-2 cells. **A** and **B**: The protein expression levels of Bax, Bcl-2 and cleaved caspase-3 in HK-2 cells incubated with KMP (10 µM) (*n* = 3). The values presented are the means ± SD of three independent experiments. NG: 5.6 mM D-glucose; M: 5.6 mM D-glucose + mannitol (24.4 mM); HG: 30 mM D-glucose; KMP: 10 µM kaempferol; KMP + HG: 10 µM kaempferol + 30mM D-glucose. ^**^P < 0.01 vs. NG group. ^##^*P* < 0.01 vs. HG group

To investigate the ability of KMP to attenuate HG-induced apoptosis, we analyzed the expression of apoptosis-related proteins. The protein expression levels of Bax, Bcl-2 and cleaved caspase-3 in HG-induced HK-2 cells were detected. Compared with NG group, the ratio of Bax/Bcl-2 and the protein expression of cleaved-caspase3 were significantly increased in HK-2 cells that were incubated with HG for 48 h (3.6-fold increase and 5.7-fold increase, respectively) (Fig. 3A and B). When the HK-2 cells in HG group were incubated with 10 µM KMP for 48 h, the ratio of Bax/Bcl-2 and relative expression of cleaved caspase-3 were reduced by 80.2% and 71.9% respectively compared with HG group (Fig. 3A and B).

### KMP influences Sirt3 expression and the activity of Akt/FoxO signaling pathway in HK-2 cells

**Fig. 4:**
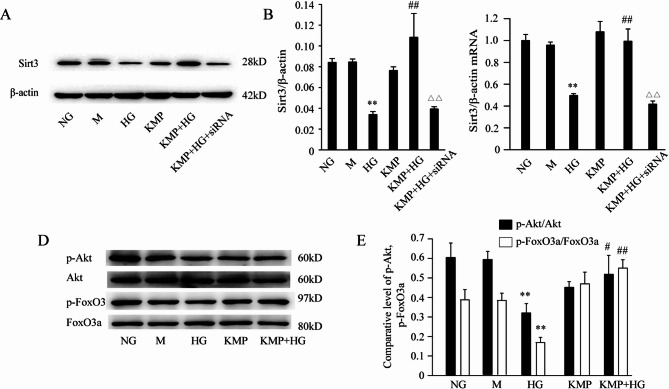
Effects of KMP on HG-induced expression of Sirt3 and Akt/FoxO3A signaling pathway in HK-2 cells. **A** and **B**: The protein expression of Sirt3 in HK-2 cells treated with 10 µM KMP under HG environment (*n* = 3). **C**: The mRNA expression of Sirt3 in KMP-incubated HK-2 cells was quantified by densitometry (*n* = 3). **D** and **E**: The protein expression levels of p-Akt, Akt, p-FoxO3a and FoxO3a were detected by Western blotting and quantified by densitometry in HK-2 cells of the normal glucose group, the mannitol group, the HG group, the KMP group and the HG plus KMP group (*n* = 3). The values presented are the means ± SD of three independent experiments. NG: 5.6 mM D-glucose; M: 5.6 mM D-glucose + mannitol (24.4 mM); HG: 30 mM D-glucose; KMP: 10 µM kaempferol; KMP + HG: 10 µM kaempferol + 30 mM D-glucose. KMP + HG + siRNA: 10 µM kaempferol + 30 mM D-glucose + Sirt3 siRNA. ^*^ *P* < 0.05 vs. NG group. ^**^ *P* < 0.01 vs. NG group. ^##^ *P* < 0.01 vs. HG group. ^∆∆^*P* < 0.01 versus HG + KMP group

To detect the influence of KMP on Sirt3 in HK-2 cells, the protein and mRNA expression levels of Sirt3 were analyzed in all groups. It can be seen in Fig. 4A and B that Sirt3 expression was reduced by 67% in HG-incubated HK-2 cells compared with NG group. Whereas, the protein and mRNA expression levels of Sirt3 were significantly increased in KMP-incubated group compared with HG group (1.45-fold increase and 1-fold increase, respectively) (Fig. 4A, B and C). To ascertain whether HG and KMP affected the activity of Akt/FoxO signaling pathway, the protein expression levels of p-Akt, Akt, p-FoxO3a, FoxO3a in all groups were detected by Western blotting. As illustrated in Fig. 4D and E, compared with the NG group, the ratio of p-Akt/Akt and p-FoxO3a/FoxO3a decreased significantly in HK-2 cells that were stimulated by HG for 48 h (0.81-fold decrease and 0.93-fold decrease, respectively). The co-incubation of HG and KMP blocked the HG-induced decrease in p-Akt and p-FoxO3a protein expression. The results in Fig. 4D and E illustrated that KMP enhanced the protein expression of p-Akt and p-FoxO3a by 0.61 times and 2.26 times respectively in comparison with HG group. As an osmotic control, mannitol did not affect the protein expression of p-Akt and p-FoxO3a.

## Discussion

The purpose of our study was to clarify the role of KMP in the progression of DN. The results suggested that KMP may have therapeutic potential in DN. The effective concentration of KMP was determined by the MTS method. Treatment with KMP not only decreased ROS generation via increasing anti-oxidative stress proteins, but also decreased apoptosis-related proteins by modulating ROS-sensitive Akt/FoxO signaling pathway.

Numerous studies have revealed that flavonoids exert a significant therapeutic effect on diabetic nephropathy primarily by modulating oxidative stress and inflammatory responses [24]. KMP, an important flavonoid, has been reported to exist in plenty of plants. The oral bioavailability of kaempferol is approximately 1.5%, which is significantly higher than that of other flavonoids [25]. Moreover, accumulating studies have suggested that KMP has a protective effect against age-related diseases through exerting its effects of anti-oxidation, anti-apoptosis, anti-inflammation and so on. The therapeutic effect of KMP on DN has been studied in this research. Firstly, the safe and effective concentration of KMP in our experiment was identified by MTS assays. Previous studies had shown that KMP possesses contradictory pharmacological effects. The anti-oxidant or pro-oxidant activity of KMP depends on its concentration. It has been reported that 50µM KMP exerted pro-oxidant and pro-apoptosis effects in glioblastoma cell, human non-small cell lung carcinoma cell line and human chronic myelogenous leukemia cell [26–28]. Consistent with these findings, our MTS results suggested that 50 µM KMP could decrease the viability of HK-2 cells significantly. Nevertheless, treatment with 10 µM or 20 µM KMP markedly reverse HG-induced inhibition of cell viability. The results indicated that the effects of KMP were significantly concentration-dependent and were most notable after 48 h. To avoid potential cytotoxicity and maximize the biological effect, we selected the concentration of 10 µM KMP in the following experiments.

Accumulating studies have demonstrated that KMP could ameliorate insulin resistance in diabetes [18], protect against doxorubicin-induced and isoproterenol-induced cardiotoxicity [29, 30], and protect SH-SY5Y cells and primary neurons from rotenone toxicity [31] owing to KMP-regulated anti-apoptotic and anti-oxidant effects. It has been well established that excessive generation of mitochondrial ROS triggered by HG represents the primary initiating mechanism in the development and progression of DN. Additionally, accumulating evidence suggests that renal tubular cell apoptosis is closely associated with the pathogenesis of DN [32]. Hence, finding compounds with potential anti-oxidative and anti-apoptotic property is necessary for counteracting hyperglycemia-caused kidney injuries. In our study, HG obviously increased oxidative stress and apoptosis-related proteins. Treatment with 10 µM KMP in HK-2 cells significantly prevented HG-induced overproduction of ROS and depletion of anti-oxidants proteins, SOD2 and catalase. Furthermore, Western blotting results revealed that a 48-hour treatment with KMP could increase Bcl-2 expression and decrease the proapoptotic proteins, Bax and cleaved caspase-3, indicating a decreased level of apoptosis in HK-2 cells. Taken together, KMP could counteract HG-induced injuries in HK-2 cells through its anti-oxidative and anti-apoptotic effects.

The major molecular mechanisms responsible for the KMP-mediated anti-oxidant effect have not been elucidated completely. Sirt3, a protein deacetylase preferentially localized in mitochondria, mediates the deacetylation of enzymes responsible for generating ROS and plays a role in several metabolic processes [33]. In addition, it has been reported that Sirt3 overexpression could upregulate the expression and activity of SOD2 and catalase in cardiomyocytes [34]. Furthermore, our recent study demonstrates that Sirt3 overexpression could significantly attenuate HG-induced oxidative stress and apoptosis in HK-2 cells [35]. In general, Sirt3 plays a critical role in protecting against ROS generation. Recently, it has been suggested that KMP could increase the expression of Sirt3 in chronic myelogenous leukemia cells [23, 28]. Based on these findings, we hypothesized that Sirt3 might contribute to the anti-oxidative effect of KMP. The obtained results showed that the treatment of KMP markedly reversed the downregulation of Sirt3 protein and mRNA expression levels induced by HG stimulation. When Sirt3 was silenced, KMP failed to prevent HG-induced suppression of cell viability and excessive ROS production. It suggested that KMP’s cytoprotection is associated with Sirt3 expression.

Forkhead box O3a (FoxO3a) plays a vital role in regulating cell survival, oxidative stress and apoptosis. When phosphorylated by the serine/threonine kinase Akt, FoxO3a underdos nucleus exclusion and relocalizes to cytoplasm. Consequently, the upregulation of p-FoxO3a could decrease the transcription of apoptosis-related proteins, such as Bim, FasL and p27 [36]. Our previous research indicateed that Sirt3 could regulate cell apoptosis via ROS-sensitive Akt/FoxO signaling pathway in HK-2 cells [35]. In this experiment, the data showed that KMP observably reversed the decrease in Sirt3 expression induced by HG. Thus, we speculated that KMP could alter the activity of Akt/FoxO signaling pathway. The Western blot experiment showed that treatment with 10µM KMP dramatically increased the ratio of p-Akt/Akt and p-FoxO3a/FoxO3a, leading to the decrease of transcriptional activity of FoxO3a and the expression of pro-apoptotic proteins. While our data demonstrate a strong association between KMP-mediated cytoprotection and Akt/FoxO3a activation, definitive causal validation through pharmacologic inhibition (e.g., Akt inhibitors) remains an essential focus for future studies.

In conclusion, the data in our experiment for the first time demonstrate that KMP exerts its anti-oxidative and anti-apoptotic effects through increasing the expression of Sirt3 and regulating ROS-sensitive Akt/FoxO signaling pathway. Our research suggests that KMP holds potential therapeutic promise for DN.

## Supplementary Information

Below is the link to the electronic supplementary material.

Supplementary Material 1

## Data Availability

The datasets used and/or analyzed during the current study are available from the corresponding author on reasonable request.

## Abbreviations

Bim: Bcl-2 interacting mediator of cell death
DCHF-DA: 5-(and-6)-chloromethyl-2′,7′-dichlorodihydrofluorescein diacetate
DN: Diabetic nephropathy
Fas-L: Fas-Ligand
FoxO: Forkhead box O
HG: High glucose
HK-2: Human kidney 2
KMP: Kaempferol
NAD: Nicotine amide dinucleotide
PVDF: Polyvinylidene difluoride
ROS: Reactive oxygen species
RTPC: Renal tubular epithelial cells
Sirt3: Sirtuin3
SOD2: Superoxide dismutase 2
