# Supplementary material for: The protective effect of kaempferol on high glucose-stimulated renal tubular epithelial cells
Source: BMC Nephrol. 2025 Aug 20;26:477. doi: 10.1186/s12882-025-04404-9 (PMC12366318; doi:10.1186/s12882-025-04404-9)

Figure 2A original：SOD2：


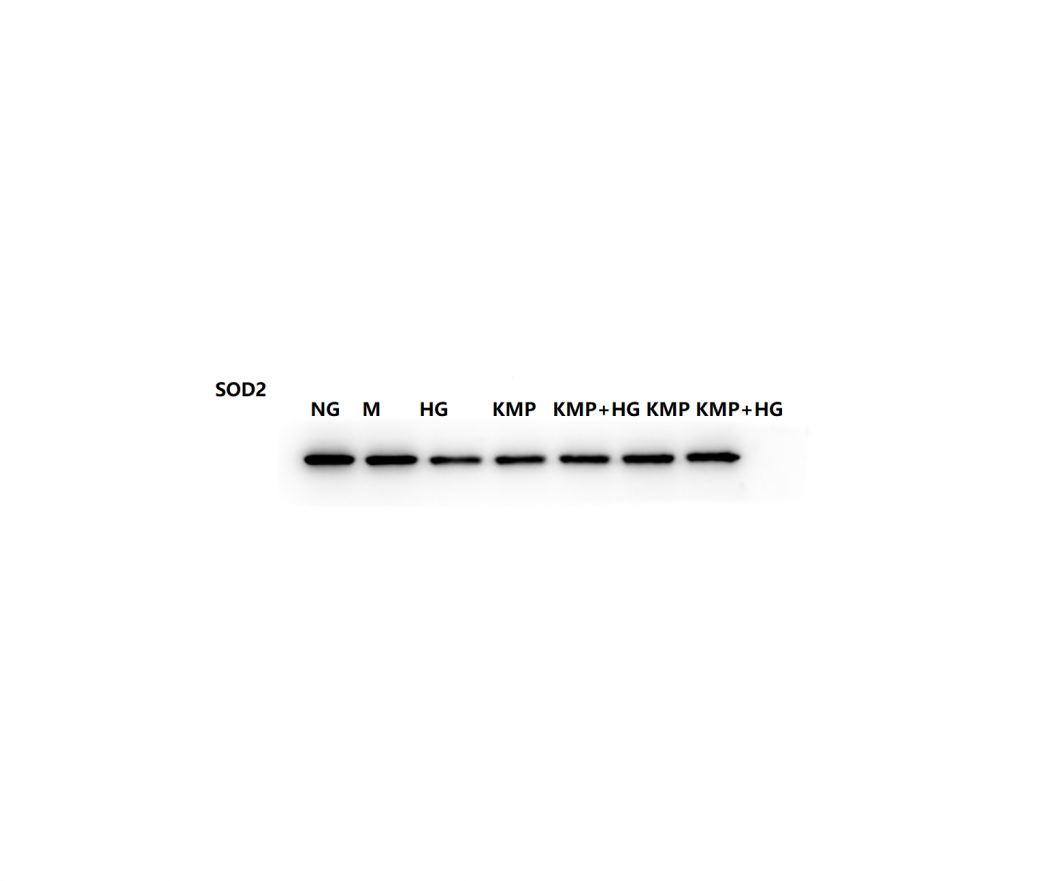


catalase：


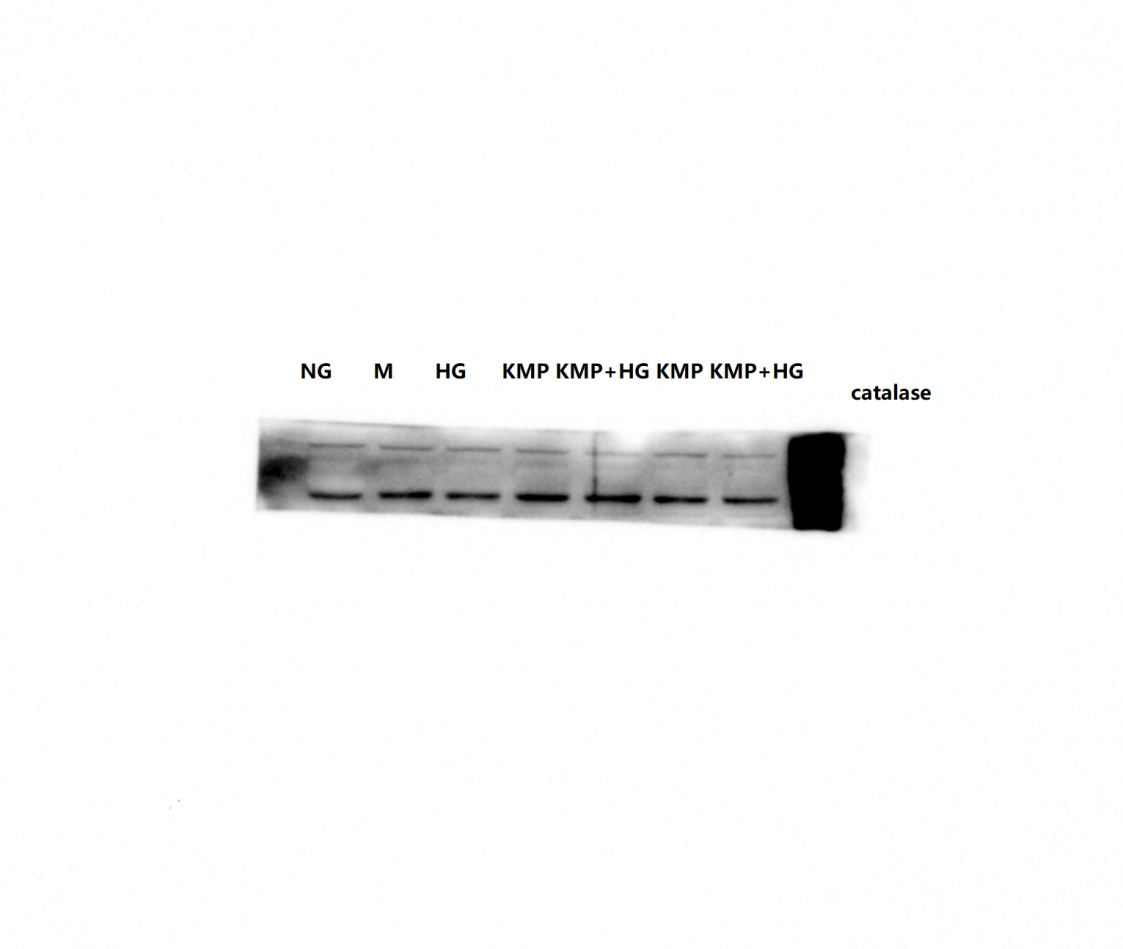


β-actin：


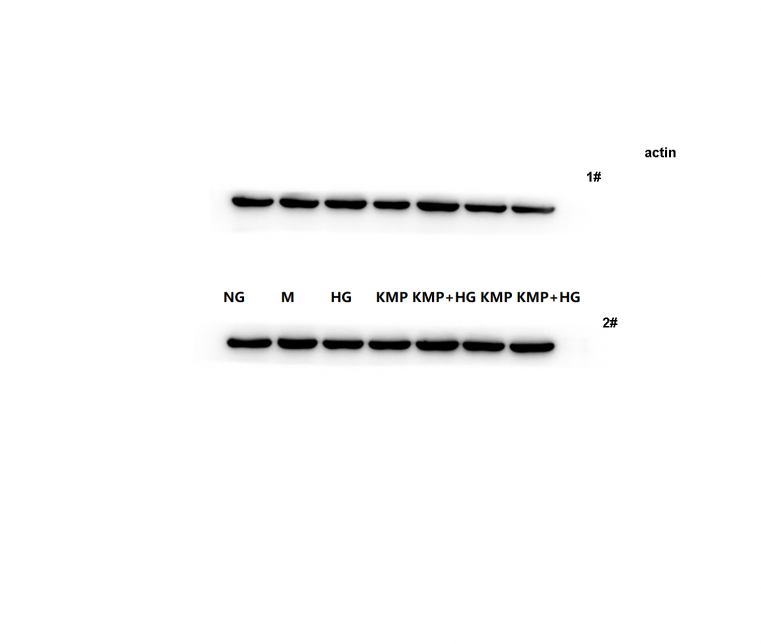


Figure 3A original：Bax：


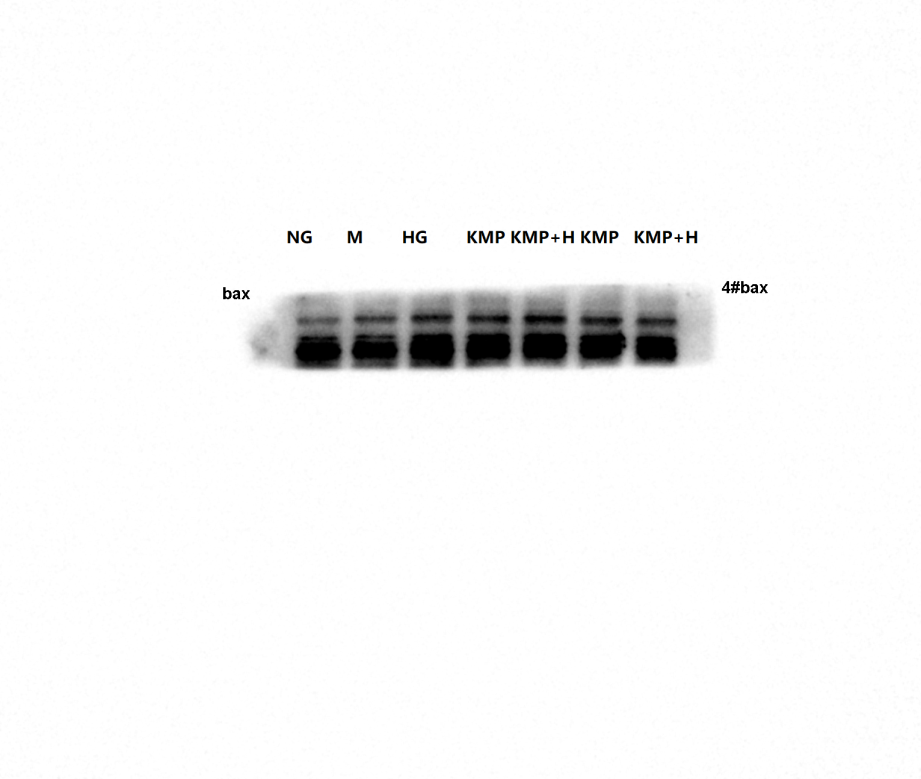


Bcl-2：


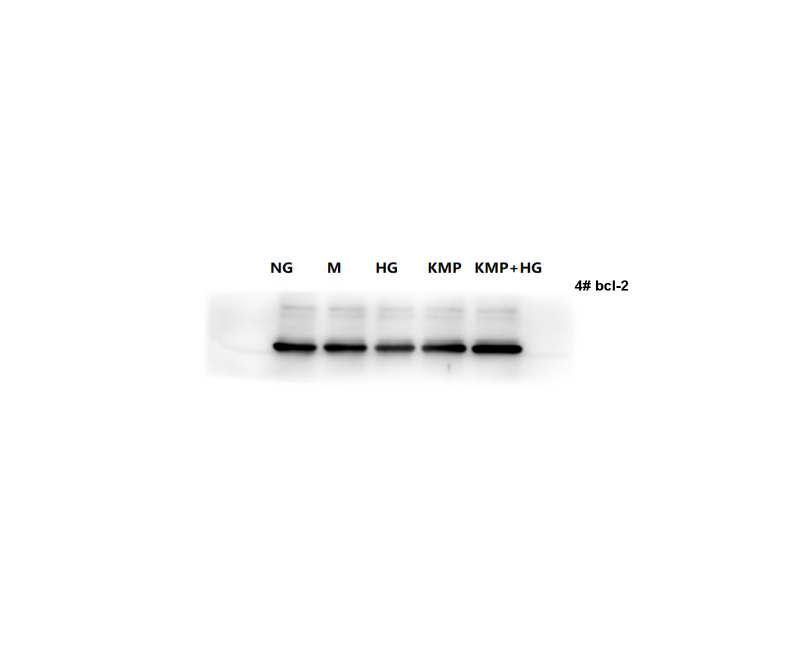


Cleaved-caspase3：


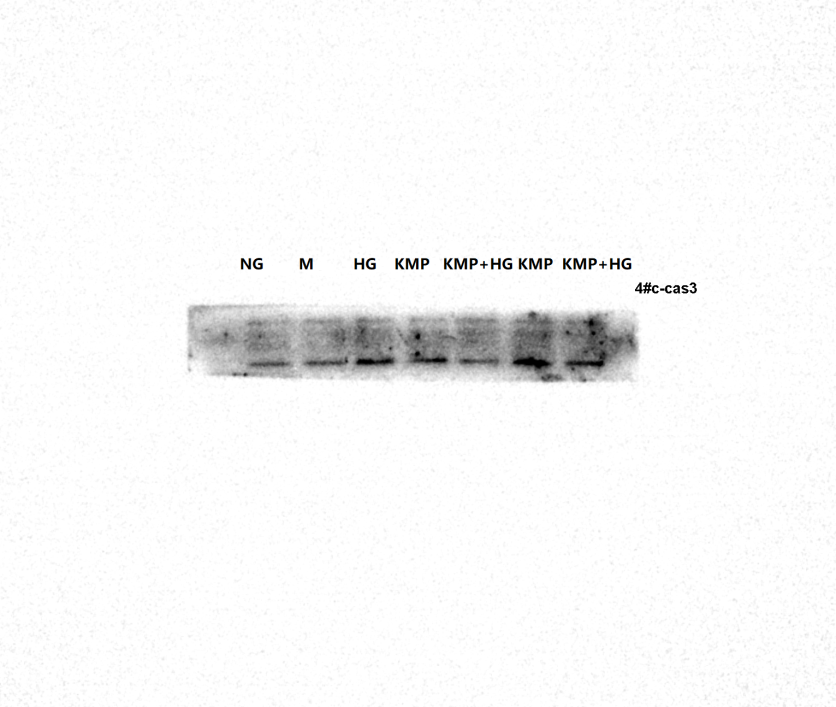


Figure 4A original：Sirt3：


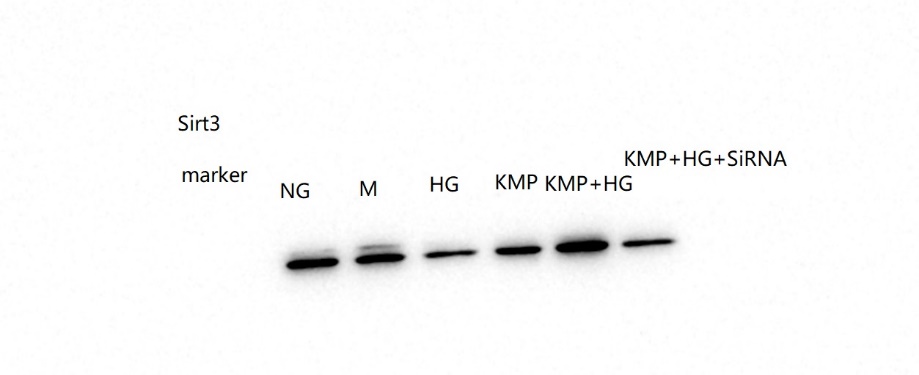


β-actin：


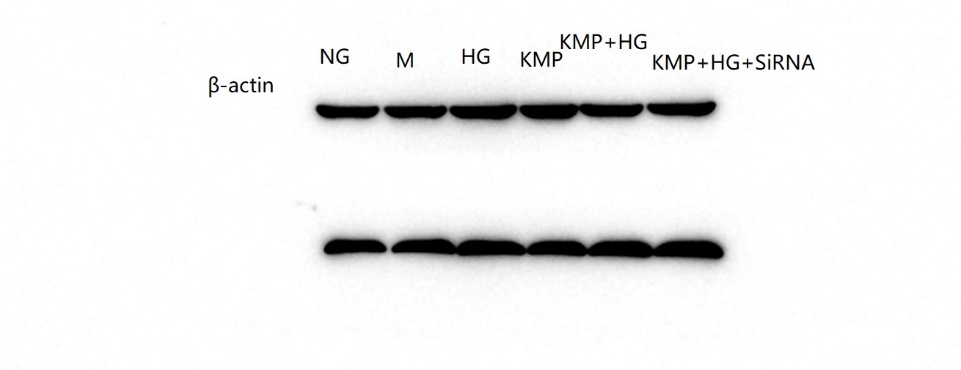


Figure 4D original：p-Akt：


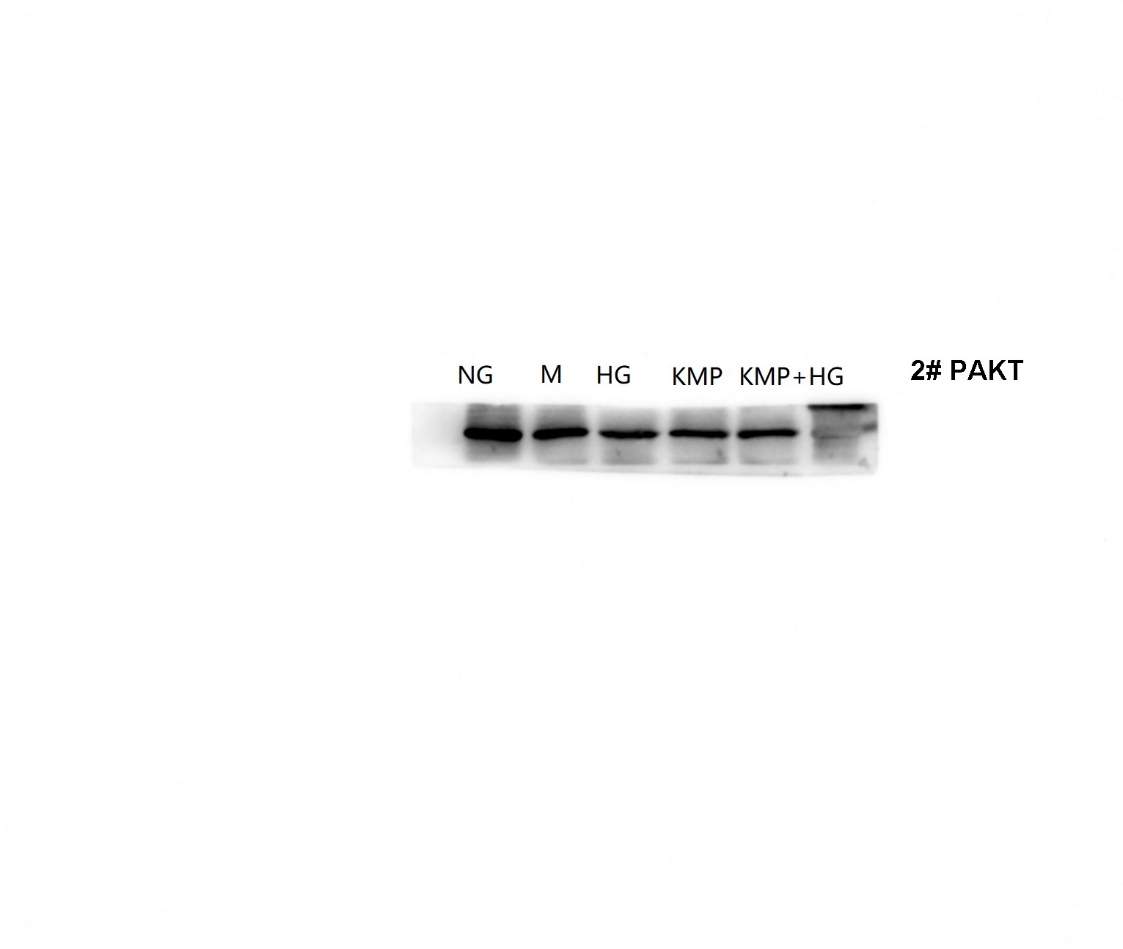


Akt：


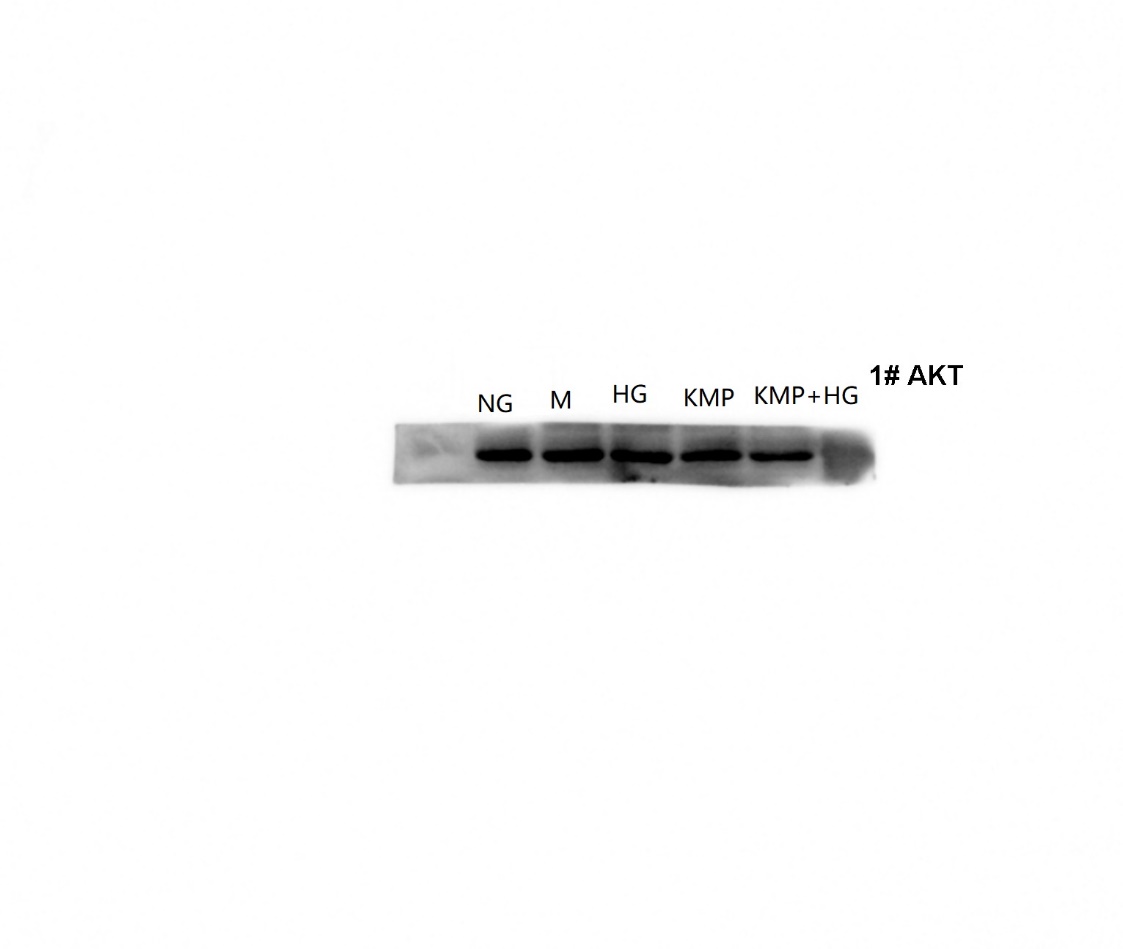


p-FoxO3a：


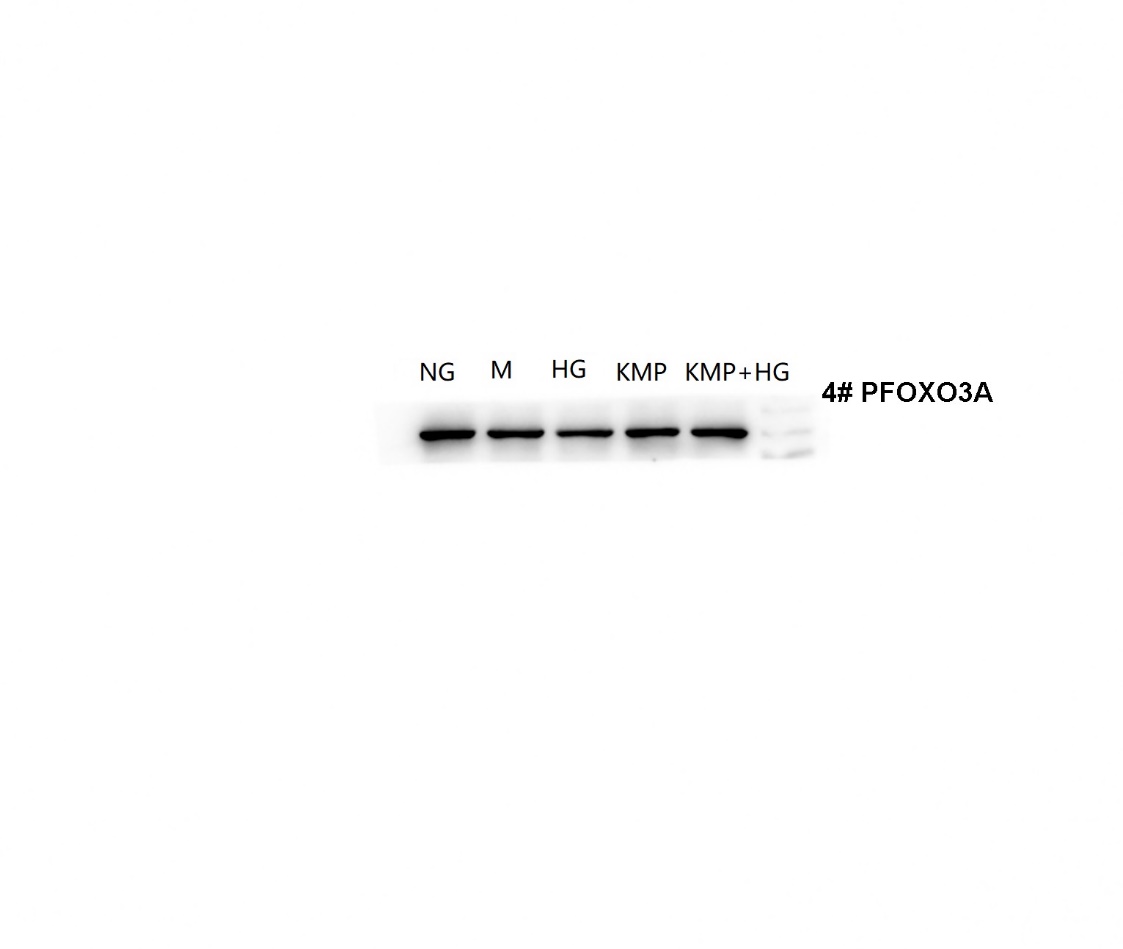


FoxO3a：


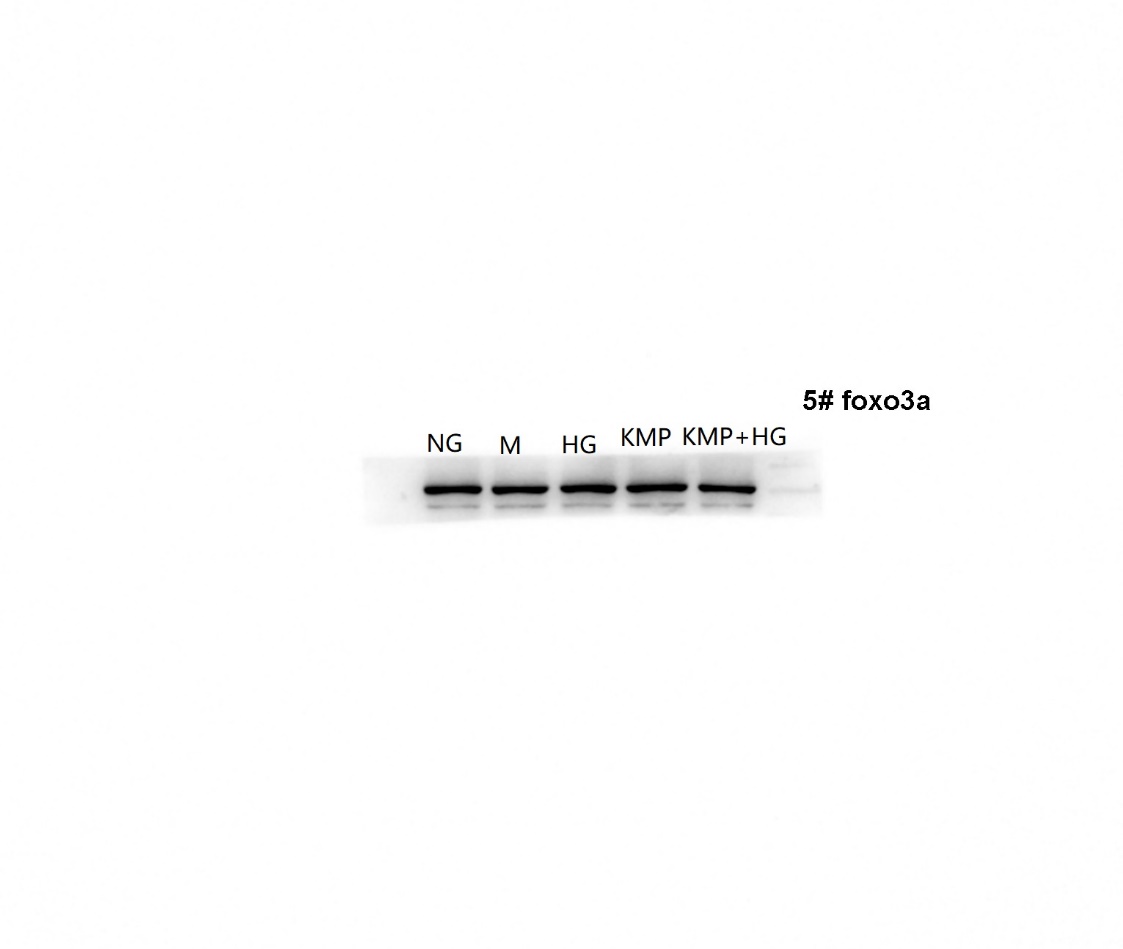

Supplement: Supplementary file 1 — Supplementary Material 1 [file 12882_2025_4404_MOESM1_ESM.docx]
